# The vaginal microbiome in HPV persistence and cervical cancer progression

**DOI:** 10.3389/fcimb.2025.1634251

**Published:** 2025-10-07

**Authors:** Jhommara Bautista, Adriana Altamirano-Colina, Andrés López-Cortés

**Affiliations:** Cancer Research Group (CRG), Faculty of Medicine, Universidad de Las Américas, Quito, Ecuador

**Keywords:** human papillomavirus, cervical cancer, vaginal microbiome, cervicovaginal dysbiosis, biomarkers and therapeutics

## Abstract

Persistent infection with high-risk human papillomaviruses (HR-HPV) is the primary cause of cervical cancer, but its progression depends on host and environmental factors beyond viral presence. The vaginal microbiome, particularly the transition from *Lactobacillus crispatus*–dominated communities to dysbiotic states enriched in *Gardnerella*, *Fannyhessea*, and *Sneathia*, has emerged as a key modulator of HPV persistence, local inflammation, and epithelial transformation. First, community state type IV (CST IV) microbiota strongly predict persistent HR-HPV infection and progression to high-grade lesions, highlighting their potential as non-invasive biomarkers for early risk stratification. Second, cervicovaginal dysbiosis alters mucosal immunity and promotes epigenetic reprogramming of both host and viral genomes, facilitating immune evasion and oncogenesis. Third, restoring *Lactobacillus* dominance through probiotics or microbial engineering holds translational promise for enhancing HPV vaccine efficacy and reducing cervical cancer burden. These findings position the vaginal microbiome not as a passive bystander, but as an active determinant of HPV-driven carcinogenesis and underscore its diagnostic and therapeutic potential in cervical cancer prevention.

## Introduction

Cervical cancer (CC) remains a significant global health challenge, ranking as the fourth most common cancer among women, with over 600,000 new cases and more than 340,000 deaths annually, disproportionately affecting low- and middle-income countries (Norenhag et al., 2024). Persistent infection with high-risk human papillomavirus (HR-HPV), particularly types 16 and 18, is recognized as the primary etiological driver of cervical carcinogenesis. While most HPV infections are transient and cleared by the immune system, a subset progresses to low- or high-grade squamous intraepithelial lesions (LSIL, HSIL) and invasive cancer. The factors underlying HPV persistence, rather than viral acquisition alone, have emerged as key determinants of disease progression and are influenced by a complex interplay between viral mechanisms, host immunity, and the local microenvironment—including the vaginal microbiome (Shen et al., 2025).

The human vaginal microbiota constitutes a dynamic and functionally diverse microbial ecosystem that plays a critical role in maintaining reproductive health and regulating mucosal immunity. A hallmark of a healthy vaginal microbiota is the dominance of *Lactobacillus* species, particularly *Lactobacillus crispatus*, which contribute to a low pH environment through lactic acid production, inhibit pathogen colonization, and support epithelial barrier integrity (Alimena et al., 2022; Norenhag et al., 2024). However, in women with HPV persistence and cervical dysplasia, a shift from Lactobacillus-dominated (Community State Type I or III) to a polymicrobial or anaerobic-dominated community (CST IV) is frequently observed. This transition is associated with reduced protective functions, increased mucosal inflammation, and the emergence of microbial taxa such as *Gardnerella vaginalis*, *Sneathia sanguinegens*, and *Fannyhessea vaginae*, which are linked to HPV persistence and dysplastic transformation (Jung et al., 2025; Lebeau et al., 2022; Norenhag et al., 2024).

Beyond bacterial communities, non-bacterial constituents of the vaginal microbiome, including viruses such as herpes simplex virus (HSV), human immunodeficiency virus (HIV), and fungi like *Candida* spp., may also modulate HPV persistence and disease progression. Co-infection with HSV-2 has been associated with increased HPV acquisition and persistence, potentially by compromising epithelial barrier integrity and promoting chronic inflammation through type I interferon dysregulation (Jones, 1995). Similarly, HIV infection is a well-documented cofactor in HPV-mediated carcinogenesis, as systemic and mucosal immunosuppression impair viral clearance and accelerate cervical lesion development (McClymont et al., 2022; Bautista and Lopez-Cortes, 2025). Moreover, HIV-infected women frequently exhibit cervicovaginal dysbiosis, which may synergize with HPV-induced immune evasion mechanisms (Kyrgiou and Moscicki, 2022). Fungal overgrowth, particularly with *Candida albicans*, has been linked to local inflammatory responses that disrupt epithelial homeostasis and may indirectly support HPV integration and persistence (Cohen et al., 2024; Valentine et al., 2025). While these interactions remain underexplored compared to bacterial dysbiosis, they highlight the need for an expanded ecological perspective on the cervicovaginal niche, considering inter-kingdom microbial dynamics as critical modulators of HPV pathogenesis.

Emerging evidence supports a bidirectional relationship between HPV infection and the vaginal microbiome. HPV not only thrives in dysbiotic environments but also contributes to the disruption of microbial homeostasis. For instance, the HPV E7 oncoprotein has been shown to suppress host defense peptides essential for Lactobacillus survival by interfering with NF-κB and Wnt/β-catenin signaling, leading to selective depletion of Lactobacilli and subsequent microbial imbalance (Lebeau et al., 2022). Such disruption impairs immune surveillance, increases oxidative stress, and facilitates immune evasion by the virus. These alterations perpetuate a microenvironment conducive to HPV persistence and promote progression from pre-neoplastic lesions to malignancy. Additionally, HPV-induced modulation of local epithelial metabolites may impact bacterial growth patterns and mucosal nutrient availability, further shaping microbial community structure (Shen et al., 2025).

High throughput sequencing technologies, including 16S rRNA gene profiling and shotgun metagenomics, have been instrumental in characterizing the vaginal microbiome and its association with cervical health. While early studies using 16S rRNA sequencing revealed broad taxonomic patterns, their limited resolution at species or strain levels hindered deeper functional insights. More recent applications of shotgun metagenomics and paired microbiome-metabolome analyses have unveiled not only species-level shifts but also differential enrichment of functional pathways in women with dysplasia. Specifically, women with LSIL and HSIL exhibit increased abundance of genes involved in nucleotide biosynthesis, peptidoglycan turnover, and inflammatory mediators, while healthy controls show enhanced capacities for amino acid synthesis and sugar metabolism-metabolic signatures that mirror microbial compositional changes and mucosal immune status (Jung et al., 2025; Norenhag et al., 2024; Shen et al., 2025).

Moreover, cervicovaginal metabolomics has revealed that women with HR-HPV infection display altered levels of key metabolites, like succinic acid and phenylacetaldehyde, linked to bacterial metabolism and immune signaling (Shen et al., 2025). The association between *Gardnerella* and increased succinic acid in cervicovaginal fluid, for example, underscores the functional consequences of microbial shifts on the local immunometabolic niche. These data provide critical insights into how vaginal microbial dysbiosis intersects with viral pathogenesis and carcinogenic signaling networks. They also highlight the value of integrated multi-omic strategies in elucidating microbial mechanisms underlying disease susceptibility and progression (Leon-Gomez and Romero, 2024; Shen et al., 2025).

Importantly, dysbiosis-associated bacterial species may exert direct oncogenic effects. *Fusobacterium nucleatum*, *Peptoniphilus lacrimalis*, and *Fannyhessea vaginae* have been implicated in promoting epithelial inflammation, DNA damage, and immune modulation, analogous to their roles in other cancers such as colorectal carcinoma (Jung et al., 2025; Norenhag et al., 2024). These species activate innate immune responses and disrupt epithelial tight junctions, creating a permissive niche for HPV persistence and neoplastic transformation. Experimental models further confirm that cervicovaginal dysbiosis exacerbates epithelial proliferation and immune suppression, accelerating HPV-driven neoplasia (Li et al., 2020; Lebeau et al., 2022).

Taken together, the vaginal microbiome is now recognized not merely as a bystander but as an active modulator of HPV persistence and cervical cancer risk. Its influence extends beyond microbial composition to encompass immune regulation, metabolic reprogramming, and epithelial-microbial crosstalk. Understanding these complex interactions offers promising avenues for early risk stratification and targeted prevention strategies. Vaginal microbiome profiling could serve as a predictive biomarker for HPV persistence and lesion progression, while therapeutic modulation, through probiotics, vaginal microbiota transplants, or metabolite-targeted interventions, may enhance mucosal resilience and reduce cervical cancer burden (Ottinger et al., 2024; Mitra et al., 2016b; Shen et al., 2025).

## Methodological advances and challenges in vaginal microbiome research

Recent methodological advances have significantly expanded our understanding of the vaginal microbiome, yet important challenges remain that limit clinical translation. Early studies primarily relied on 16S rRNA gene sequencing due to its cost-effectiveness and capacity to classify bacterial taxa at the genus level. However, this approach lacks resolution for strain-level discrimination and provides limited insights into microbial function. Moreover, primer bias, low coverage of non-bacterial domains (e.g., fungi, viruses), and poor sensitivity to detect low-abundance but clinically relevant microbes further constrain its utility in cervical cancer research (Li et al., 2024; Di Paola et al., 2017; Tango et al., 2020). These limitations have spurred interest in shotgun metagenomic sequencing, which offers higher resolution, enabling identification of species and functional gene pathways associated with disease progression. For instance, shotgun metagenomics has revealed that high-risk HPV (hrHPV) persistence correlates with enrichment in microbial pathways involved in folate biosynthesis and oxidative phosphorylation—features undetectable via 16S sequencing alone. Nonetheless, the high cost, bioinformatic complexity, and requirements for large sample sizes and high-quality DNA still hinder routine application in large cohorts, especially in low-resource settings (Li et al., 2024; Jung et al., 2025; Tango et al., 2020).

Community State Type (CST) profiling represents another methodological advancement, categorizing vaginal microbial compositions into distinct clusters dominated by *Lactobacillus* sp*ecies* (e.g., CST I–III, V) or characterized by high microbial diversity and dysbiosis (CST IV). CST classification is clinically informative: CST IV, marked by reduced *Lactobacillus* and higher diversity of anaerobes, is consistently associated with HPV persistence, CIN progression, and heightened cervical cancer risk (Shen et al., 2025; Mitra et al., 2020; Molina et al., 2022b). However, challenges persist in standardizing CST definitions across studies due to methodological variability in sequencing protocols, thresholds for relative abundance, and sample collection timing. For instance, longitudinal analysis shows that CSTs are not static; rather, they fluctuate with hormonal cycles, sexual activity, antibiotic exposure, and immune status (Xu et al., 2022; Molina et al., 2022b), necessitating temporal sampling strategies to capture dynamic microbial shifts. Furthermore, while CST profiling helps stratify risk, it does not account for strain-specific functional differences, such as *Lactobacillus crispatus* versus *L. iners*, which differ in their ability to maintain a protective acidic environment and modulate mucosal immunity (Di Paola et al., 2017; Wang et al., 2022a).

Multi-omics integration has emerged as a frontier approach to overcome the limitations of single-modality studies. By combining metagenomics, metatranscriptomics, metabolomics, and host epigenetic profiling, researchers aim to unravel the complex host-microbiome interactions that underlie carcinogenesis. For instance, microbial metabolites such as lactic acid, short-chain fatty acids (SCFAs), and indole derivatives have been linked to modulation of host immunity and HPV clearance (Shen et al., 2025; Jung et al., 2025). Integrative omics has also identified microbial genes such as *ABCG2*, *TDG*, and *PCNA*, expressed in cervical microbial communities, as predictive biomarkers for CIN progression, enabling machine learning models such as random forest classifiers to distinguish between benign and high-grade lesions (Wang et al., 2022a). However, these multi-omics studies require large, well-annotated biobanks, standardized sampling procedures, and sophisticated computational pipelines for data normalization and interpretation. Inconsistencies in sample processing, like swab versus biopsy, differences in DNA extraction kits, or lack of matched host transcriptome data, can introduce batch effects that confound biological signals (Li et al., 2024; Musa et al., 2023).

Standardization and reproducibility represent additional challenges. There is a lack of consensus on optimal methods for sampling, storage, and sequencing. For instance, different anatomical sites (vaginal vault, ectocervix, endocervix) harbor distinct microbial communities, and failure to control for this variability limits inter-study comparability (Xu et al., 2022; Mitra et al., 2020). Storage conditions, such as temperature and preservatives, affect microbial integrity, especially in low biomass environments like the cervix. Moreover, many studies use small, cross-sectional cohorts, limiting statistical power and generalizability across populations. Population-specific factors, such as ethnicity, diet, sexual practices, and access to healthcare, also influence microbiome composition and are often underreported or inconsistently controlled (Jung et al., 2025; Molina et al., 2022b; Tango et al., 2020). Consequently, large, longitudinal, multi-center studies with harmonized protocols are needed to validate microbial biomarkers for cervical disease and assess their performance across diverse settings.

Lastly, despite growing evidence of the microbiome’s role in cervical carcinogenesis, most research has been observational, lacking mechanistic validation. *In vitro* models (e.g., cervical epithelial cell co-culture with microbiota) and *in vivo* murine models colonized with human vaginal microbiota are emerging tools to elucidate causal relationships (Musa et al., 2023). However, ethical and biological limitations, like differences between human and murine vaginal physiology, complicate translation. Functional studies are also limited by the difficulty in isolating and culturing fastidious or anaerobic cervicovaginal microbes. Advances in culturomics and organoid co-culture models may help bridge this gap. Ultimately, the integration of methodological rigor with translational focus is essential to move vaginal microbiome research from correlative association to actionable clinical interventions, such as microbial risk stratification, personalized probiotics, and targeted microbiota modulation for HPV persistence or cervical cancer prevention (Jung et al., 2025; Molina et al., 2022b; Wang et al., 2022a).

## The role of vaginal microbiome composition in HPV persistence and cervical carcinogenesis

**Figure 1:**
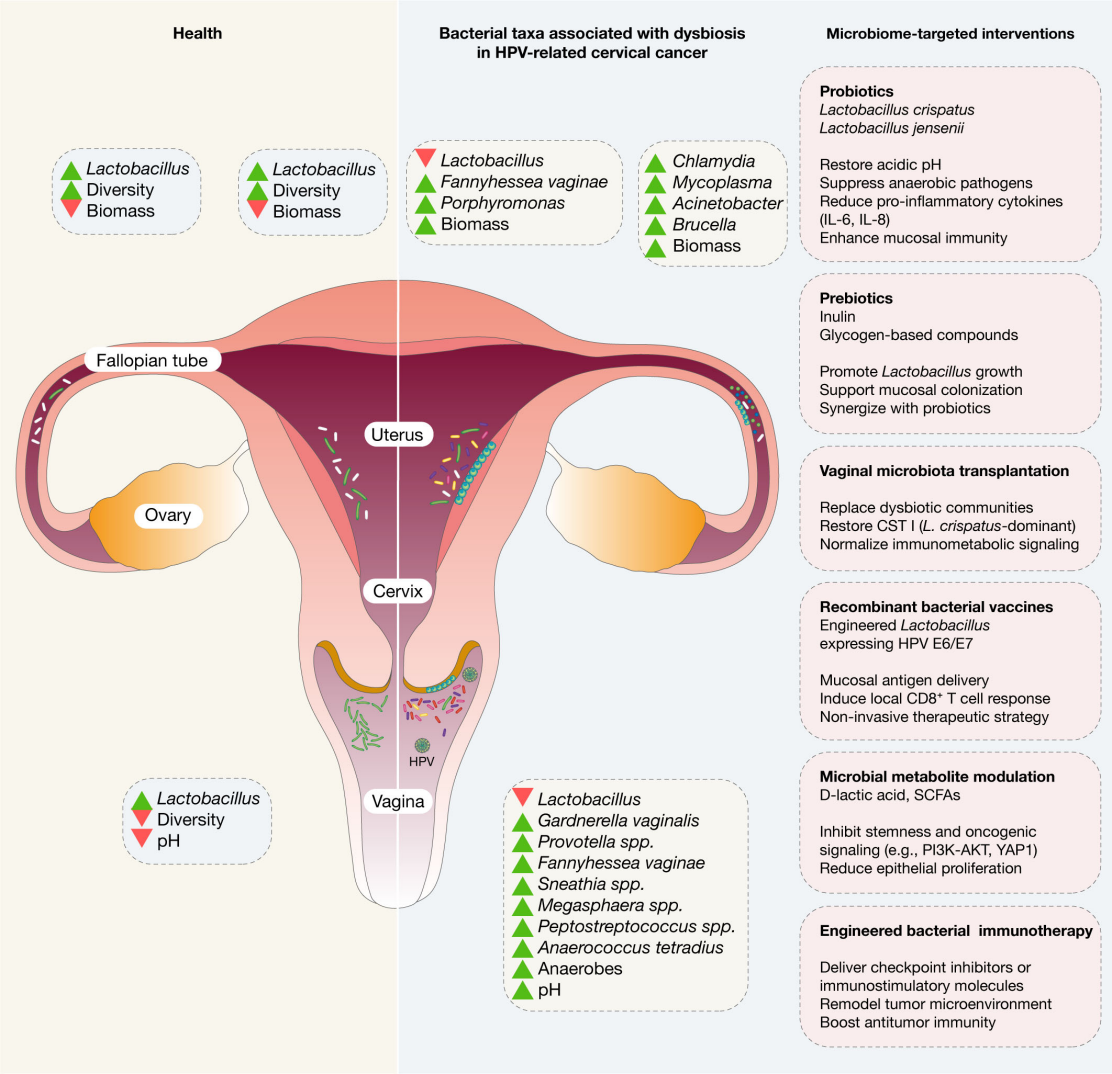
Microbial profiles across the female reproductive tract in health and during HPV persistence and cervical cancer progression. Microbial communities along the female reproductive tract (FRT) display distinct spatial distributions and respond dynamically to both physiological and pathological states. In healthy reproductive-age women, the lower FRT, comprising the vagina and cervix, hosts the greatest microbial density. These sites are typically dominated by *Lactobacillus* species, which maintain low pH through lactic acid secretion, contributing to mucosal barrier integrity and defense against pathogens. In disease states, particularly in the setting of persistent high-risk HPV infection and cervical cancer, the lower FRT microbiome undergoes significant dysbiosis. *Lactobacillus* depletion is frequently observed, accompanied by an overrepresentation of pro-inflammatory anaerobic taxa such as *Sneathia*, *Gardnerella*, *Fannyhessea vaginae, Prevotella*, *Megasphaera*, *Anaerococcus*, *and Peptostreptococcus*. These changes are associated with elevated vaginal pH, impaired epithelial defenses, and heightened local inflammation, all of which may favor virus persistence and neoplastic transformation. Collectively, these alterations suggest that microbiota may influence not only the susceptibility to and persistence of HPV infection but also the progression and treatment response of gynecological malignancies. Additionally, the microbiome-targeted interventions are encompassed by probiotics, prebiotics, vaginal microbiome-transplantation, recombinant bacterial vaccines, microbial metabolite modulation, and engineered bacterial immunotherapy.

Persistent infection with HR-HPV is a necessary but insufficient condition for CC development. An increasing body of evidence points to the cervicovaginal microbiota as a key modifier of HPV persistence and subsequent carcinogenic progression. In healthy women, the vaginal microbiome is typically dominated by *Lactobacillus* species, particularly *L. crispatus*, *L. iners*, *L. gasseri*, and *L. jensenii*, which maintain a low vaginal pH through lactic acid production and provide protection via bacteriocins and competitive exclusion of pathogens (Łaniewski et al., 2020b; Cheng et al., 2020). This *Lactobacillus*-dominant community state (CST I–III, V) is associated with lower HPV acquisition risk and faster viral clearance (Mitra et al., 2015). In contrast, HPV-infected women frequently exhibit a shift toward a non-*Lactobacillus*-dominant CST IV microbiome, characterized by increased diversity and abundance of anaerobic bacteria such as *Gardnerella*, *Prevotella*, *Fannyhessea vaginae*, *Sneathia*, *Megasphaera*, and *Peptostreptococcus* (Mitra et al., 2016a; Sharifian et al., 2023; Mitra et al., 2015) (**Figure 1**). These dysbiotic microbial communities elevate vaginal pH, disrupt mucosal barriers, and generate inflammatory microenvironments that may promote viral persistence and epithelial transformation (Lebeau et al., 2022; Norenhag et al., 2024). For instance, *Sneathia sanguinegens* and *Anaerococcus tetradius* are significantly enriched in high-grade squamous intraepithelial lesions (HSIL) and CC, while *L. jensenii* levels decline with disease severity (Mitra et al., 2015, 2016a).

Next-generation sequencing and 16S rRNA gene profiling have confirmed that women with CIN2/3 or invasive CC exhibit significantly higher alpha-diversity and lower *Lactobacillus* abundance than healthy controls (Łaniewski et al., 2020b; Kudela et al., 2021; Mitra et al., 2016a). The transition from a protective, *Lactobacillus*-rich ecosystem to a dysbiotic and pro-inflammatory milieu is thought to facilitate HPV integration and immune evasion. Furthermore, the presence of *L. iners*, while still a *Lactobacillus* species, has been paradoxically associated with HPV persistence and oncogenic progression due to its less stable and potentially pro-inflammatory metabolic profile. Population-specific studies have further highlighted racial and ethnic disparities in vaginal microbiome composition and CC risk. For example, Latina and African-American women display a higher prevalence of non-*Lactobacillus*-dominant microbiota, partially explaining the disproportionate burden of CC observed in these populations (Mancilla et al., 2024).

The composition of the vaginal microbiome plays a pivotal role in modulating host susceptibility to high-risk HPV persistence and the progression to cervical cancer. Dysbiotic profiles enriched in *Gardnerella vaginalis*, *Fannyhessea vaginae*, and *Dialister* species have been consistently associated with hrHPV infection and the development of SIL and CIN (Pai et al., 2025; Bridy et al., 2025; Molina et al., 2024). Longitudinal studies confirm that persistent CST IV profiles are more likely to promote neoplastic transformation than stable CST I communities (Huang et al., 2024; Bridy et al., 2025). Mechanistically, these dysbiotic states disrupt mucosal defenses, impair immune activation, and generate pro-tumorigenic metabolites such as succinate and indole derivatives, creating an optimal environment for viral persistence and integration (Liu et al., 2022; Myeong et al., 2025).

The impact of microbial metabolites extends to the regulation of cervical stem cells and early oncogenic pathways. D-lactic acid produced by *L. crispatus* and *L. jensenii* suppresses YAP1 signaling and stem cell renewal, providing anti-cancer effects, whereas L-lactic acid, predominant in *L. iners*, lacks such activity (Myeong et al., 2025). The overgrowth of *Gardnerella* and *Fannyhessea vaginae* may counteract these protective effects, promoting inflammatory and proliferative cues in the cervical epithelium (Bridy et al., 2025; Molina et al., 2024). Shifts in community state composition have been shown to precede cytological abnormalities, suggesting a temporal link between microbiome transition and lesion development (Huang et al., 2024; Ilhan et al., 2019; Molina et al., 2024).

Sociodemographic factors also shape microbial patterns and disease susceptibility. CST IV is more prevalent among Black and Latina women, even when *Lactobacillus* is dominant, suggesting that not all CST I profiles offer equal protection, possibly due to host–microbe interactions or immune background (Ilhan et al., 2019; Tossas et al., 2023). Intermediate CSTs, such as those dominated by *L. iners*, are less stable and more likely to transition toward a dysbiotic state, thereby increasing the risk of hrHPV persistence and progression (Huang et al., 2024; Liu et al., 2022; Tossas et al., 2023).

Overall, the interplay between vaginal microbiota and HPV reflects a dynamic and bidirectional relationship. Dysbiosis not only facilitates HPV persistence and cervical neoplasia but may also be perpetuated by viral-induced alterations in the epithelial and immune landscape. These findings underscore the potential of microbiota profiling as a diagnostic biomarker and therapeutic target for HPV-associated cervical disease (Wang et al., 2023; Kudela et al., 2021; Sharifian et al., 2023), and are further reinforced by mechanistic and longitudinal microbiome data (Pai et al., 2025; Myeong et al., 2025; Molina et al., 2024).

## Community state type profiling predicts HPV persistence and cervical cancer risk

The classification of vaginal microbiota into CSTs has emerged as a powerful framework to predict human papillomavirus (HPV) persistence and the progression to cervical intraepithelial neoplasia (CIN) and cervical cancer. CST profiling categorizes vaginal microbial communities into discrete groups based on dominant bacterial taxa, often centered around Lactobacillus species. Five primary CSTs have been identified: CST I (*Lactobacillus crispatus*-dominated), CST II (*L. gasseri*), CST III (*L. iners*), CST V (*L. jensenii*), and CST IV, which lacks a dominant *Lactobacillus* and is characterized by high microbial diversity and abundance of anaerobic bacteria such as *Gardnerella, Fannyhessea vaginae*, and *Prevotella* (Ottinger et al., 2024; Jung et al., 2025; Munoz Briones and Brubaker, 2025). Among these, CST IV is consistently associated with a higher risk of HPV persistence, lower rates of viral clearance, and progression to high-grade cervical lesions. Longitudinal studies have revealed that women with CST IV at baseline are significantly more likely to harbor persistent high-risk HPV infections and exhibit lower regression rates of CIN 2/3 lesions (Kero et al., 2023; Molina et al., 2022a; Andralojc et al., 2021).

Mechanistically, CST IV is linked to elevated vaginal pH, impaired mucosal immunity, and pro-inflammatory cytokine profiles, creating a permissive environment for HPV persistence and neoplastic transformation. In contrast, CST I, dominated by *L. crispatus*, is considered protective due to its robust production of lactic acid, maintenance of low vaginal pH, and modulation of anti-viral host immune responses. Notably, *L. iners*-dominant CST III occupies an intermediate role; while it maintains some lactic acid production, *L. iners* lacks many of the immunomodulatory and barrier-enhancing properties of *L. crispatus* and is frequently associated with transitions to CST IV under perturbation, such as antibiotic use or hormonal changes (Bommana et al., 2022; Jung et al., 2025; Berman et al., 2020). These ecological dynamics highlight the instability of non-optimal CSTs and their association with mucosal vulnerability to HPV.

Recent advances in multi-omic analyses have refined CST-based predictions by integrating microbial composition with functional capacity and host response. For example, metagenomic and metabolomic profiling have identified that CST IV communities are enriched in metabolic pathways related to polyamine biosynthesis and nitrate reduction, pathways implicated in inflammation and epithelial proliferation, further reinforcing their oncogenic potential (Andralojc et al., 2021; Munoz Briones and Brubaker, 2025; Lee et al., 2023). In a systems biology framework, CSTs represent not only taxonomic clusters but functional ecosystems whose metabolites and immunological interactions influence the trajectory of HPV infection. Integrative models using machine learning classifiers trained on CST-associated taxonomic and metabolic features have achieved high predictive accuracy in distinguishing persistent versus transient HPV infections (Virtanen et al., 2017; Munoz Briones and Brubaker, 2025).

Moreover, CST classification has significant implications for vaccine responsiveness and therapeutic interventions. Studies suggest that women with *Lactobacillus*-depleted CST IV profiles may exhibit attenuated mucosal immune responses to HPV vaccination, potentially due to altered antigen presentation and inflammatory milieu. This observation underscores the importance of stratifying patients by CST in clinical trials and considering microbiome restoration as an adjunct to vaccination strategies (Kero et al., 2023; Andralojc et al., 2021). Similarly, emerging microbiome-modulating therapies, including probiotics, vaginal microbiota transplantation (VMT), and bacteriophage cocktails, aim to convert high-risk CST IV profiles into protective Lactobacillus-dominant CSTs, thereby restoring mucosal homeostasis and promoting HPV clearance (Molina et al., 2022a; Berman et al., 2020; Lee et al., 2023).

However, despite the translational promise of CST profiling, important limitations remain. CSTs are not static; they fluctuate with menstrual cycle phase, sexual activity, contraceptive use, and age. Cross-sectional studies may misclassify transient states or fail to capture meaningful transitions that precede HPV acquisition or clearance. Longitudinal designs and time-series sampling are therefore essential to elucidate causal relationships between CST transitions and cervical disease dynamics (Bommana et al., 2022; Ottinger et al., 2024; Berman et al., 2020). Additionally, variation in CST definitions across studies, driven by differences in sequencing depth, clustering thresholds, and reference databases, complicates meta-analysis and hampers reproducibility. There is an urgent need for harmonized CST nomenclature and standardization in bioinformatic pipelines to ensure consistency across research and clinical settings (Jung et al., 2025; Virtanen et al., 2017).

Lastly, CSTs may oversimplify the complexity of vaginal microbiota by focusing on dominant taxa while ignoring rare but functionally significant microbes. For instance, CST IV encompasses a diverse range of anaerobic communities with distinct immunological footprints, yet current classification systems treat them as a single dysbiotic category. To address this, novel sub-classifications and unsupervised clustering approaches are being developed to capture finer ecological nuances. Integration with host transcriptomic and proteomic data may also help identify specific CST subtypes that are most predictive of HPV persistence or CIN progression (Virtanen et al., 2017; Munoz Briones and Brubaker, 2025). The development of predictive models such as Latent Interacting Variable-Effects (LIVE) modeling, which integrates CSTs with multi-omic and clinical variables, represents a step toward precision diagnostics in cervical cancer prevention (Andralojc et al., 2021; Munoz Briones and Brubaker, 2025).

In summary, CST profiling provides a robust yet evolving framework to stratify HPV-related cervical cancer risk based on vaginal microbial ecology. While CST I confers protective effects through dominance of beneficial *Lactobacillus* species, CST IV represents a high-risk state characterized by dysbiosis, inflammation, and impaired viral clearance. Ongoing efforts to refine CST definitions, integrate functional omics, and apply systems biology approaches will enhance the predictive power and clinical utility of this classification, paving the way for microbiome-based interventions in cervical cancer prevention and management (Ottinger et al., 2024; Andralojc et al., 2021; Lee et al., 2023).

## Microbiome-driven modulation of local immune responses in the cervicovaginal environment

The vaginal microbiome plays a pivotal role in orchestrating mucosal immune responses that influence HPV persistence and cervical carcinogenesis. A *Lactobacillus*-dominant microbiota, particularly *Lactobacillus crispatus*, maintains immune homeostasis through multiple mechanisms, including pH regulation, antimicrobial peptide production, and modulation of host immune signaling pathways (Shen et al., 2024; Decout et al., 2024). These protective effects are mediated in part by surface layer proteins (SLPs) of *L. crispatus*, which shield innate immune receptors and selectively interact with the anti-inflammatory receptor DC-SIGN, thereby attenuating NF–κB–mediated cytokine release (Decout et al., 2024). Conversely, depletion of *Lactobacillus* and expansion of anaerobic bacteria such as *Gardnerella vaginalis*, *Prevotella* spp., and *Sneathia* spp. leads to mucosal immune dysregulation. These non-optimal microbial profiles are associated with upregulated Toll-like receptor (TLR) signaling, especially via TLR4, resulting in elevated pro-inflammatory cytokine production (e.g., IL-1β, IL-6, IL-8, TNF-α) by local antigen-presenting cells and epithelial cells (Wang et al., 2022b; Anahtar et al., 2015). The resulting inflammation compromises epithelial integrity and may promote HPV persistence and neoplastic progression through chronic immune activation and impaired antiviral defenses (Anahtar et al., 2015; Kudela et al., 2021; Cicchini et al., 2016).

Evidence from clinical and translational studies supports the immunomodulatory influence of cervicovaginal dysbiosis. For instance, women with high-diversity microbiota and low *Lactobacillus* abundance exhibit elevated genital cytokine levels and recruitment of activated leukocytes, contributing to a pro-inflammatory milieu that favors viral persistence and progression to CIN (Anahtar et al., 2015; Chan et al., 2022). This is further compounded by the HPV-mediated suppression of specific chemokines like CXCL14, which impairs immune cell trafficking and antitumor responses (Cicchini et al., 2016). Moreover, cytokine profiles in dysbiotic states vary across stages of disease. For example, increased levels of IL-36γ, IL-6, and IL-8 have been identified in invasive cervical carcinoma and are correlated with *Sneathia* and other BV-associated species, suggesting a microbial contribution to immune evasion and tumor-promoting inflammation (Shen et al., 2024) (Elovitz et al., 2019). Notably, cervicovaginal immune tone is not solely determined by microbial composition, but also by microbial function, such as the production of immune-modulatory metabolites and interactions with mucosal receptors (Wang et al., 2022b; Decout et al., 2024). The CVM exerts a profound immunological influence in the cervicovaginal environment. Dysbiosis undermines mucosal defenses and facilitates persistent HPV infection through the induction of local inflammation, suppression of chemokine-mediated immune recruitment, and epithelial barrier disruption. Restoring *Lactobacillus* dominance, particularly *L. crispatus*, represents a promising strategy to recalibrate local immune responses and prevent HPV-associated cervical pathology (Decout et al., 2024; Kudela et al., 2021).

Emerging evidence suggests that the gastrointestinal and vaginal microbiomes are not isolated ecosystems, but instead communicate via a bidirectional gut–vagina axis involving microbial metabolites, systemic immune signaling, and, in some cases, the translocation of microbial components. Gut dysbiosis, particularly depletion of *Lactobacillus* and enrichment of pro-inflammatory taxa, has been linked to systemic inflammation and mucosal immune dysregulation, which may indirectly influence the cervicovaginal environment (Amabebe and Anumba, 2020). Metabolites produced by gut microbes, such as SCFAs including butyrate and acetate, modulate dendritic cell maturation, epithelial barrier function, and cytokine release, thereby shaping immune responses at distal mucosal sites, including the cervix (Sun et al., 2017). Shared microbial taxa such as *Lactobacillus* and *Bifidobacterium* are commonly found in both gut and vaginal niches, especially during early life, and may participate in immune imprinting and mucosal tolerance (McCauley et al., 2022). These interconnections highlight a systemic dimension to mucosal microbiome interactions and suggest that gut dysbiosis may predispose to HPV persistence via immune modulation at the cervicovaginal interface. Nevertheless, mechanistic studies directly linking gut microbial alterations to HPV-associated cervical pathogenesis remain limited and warrant further investigation.

## Epigenetic modifications induced by the vaginal microbiome in HPV-related cervical lesions

Persistent infection with HR-HPVs, particularly types 16 and 18, is necessary for CC. However, the transformation of infected epithelium into malignant lesions also requires a permissive host microenvironment, including inflammatory and epigenetic reprogramming processes influenced by the vaginal microbiota (Huang et al., 2024; Łaniewski et al., 2020b). Emerging evidence suggests that microbial dysbiosis within the cervicovaginal niche promotes epigenetic alterations in both host and viral genomes. These changes include DNA methylation of tumor suppressor genes, histone modifications, and the deregulation of non-coding RNAs, which jointly impair immune surveillance and enable oncogenic progression (Da Silva et al., 2021). For example, high-diversity microbial communities, often enriched in *Gardnerella*, *Prevotella*, and *Fannyhessea vaginae*, can trigger chronic inflammation and oxidative stress. These processes increase DNA damage and induce aberrant methylation patterns in epithelial cells, particularly in promoter regions of key regulatory genes (Huang et al., 2024).

A critical mechanism involves HPV oncoproteins E6 and E7, which directly interact with the host epigenetic machinery. E6 promotes degradation of p53, while E7 inactivates pRb, thereby facilitating uncontrolled proliferation. Concomitantly, these oncoproteins can induce the overexpression of histone-modifying enzymes and DNA methyltransferases (DNMTs), enhancing gene silencing at tumor suppressor loci such as *CDKN2A* and *DAPK1* (Da Silva et al., 2021; Castro-Oropeza and Piña-Sánchez, 2022). Notably, *CXCL14*, a chemokine crucial for immune cell recruitment, is frequently silenced in HPV-positive CCs through E7-driven promoter hypermethylation, leading to immune evasion (Cicchini et al., 2016). The epigenetic footprint of HPV infection can be detected even in early stages of CIN, particularly when coupled with microbial dysbiosis. For instance, a recent methylome-wide study found that women with low-grade lesions and HR-HPV infection exhibited a distinctive signature of DNA methylation termed the WID-HPV index, which was absent in high-grade lesions or cancer, possibly reflecting an early, abortive immune response that fails during progression (Herzog et al., 2023). Furthermore, the interplay between vaginal microbiota and host chromatin state may shape HPV latency and reactivation. Microbial metabolites and inflammation-related signaling pathways modulate access to transcription factors and chromatin remodelers, subtly reshaping the epigenetic landscape in favor of HPV persistence and oncogenesis (Castro-Oropeza and Piña-Sánchez, 2022). Lastly, the vaginal microbiome contributes to HPV-related cervical carcinogenesis not only by modulating immune and inflammatory pathways but also through epigenetic reprogramming of host and viral genomes. These findings underscore the potential of microbiome-epigenome axes as biomarkers for early detection and targets for therapeutic intervention in HPV-associated cervical lesions (Huang et al., 2024; Łaniewski et al., 2020b; Castro-Oropeza and Piña-Sánchez, 2022).

## Impact of vaginal microbiota alterations on HPV vaccine efficacy

Although prophylactic vaccines such as Gardasil and Cervarix have significantly reduced the incidence of HPV infection and related cervical neoplasms, growing evidence suggests that the composition of the vaginal microbiome may influence vaccine efficacy and immune responsiveness (Lin et al., 2010; Amiri et al., 2025). A healthy cervicovaginal environment, typically dominated by *Lactobacillus crispatus*, supports mucosal immune homeostasis by maintaining low pH, suppressing inflammation, and enhancing barrier integrity. In contrast, dysbiosis, marked by depletion of *Lactobacilli* and enrichment of anaerobic taxa such as *Gardnerella*, *Prevotella*, and *Sneathia*, has been associated with reduced HPV clearance and persistent infection, which may compromise vaccine-mediated protection (Huang et al., 2024; McClymont et al., 2022). One proposed mechanism is that vaginal dysbiosis disrupts the cytokine milieu and antigen-presenting cell function, both of which are crucial for initiating robust adaptive responses following vaccination. A recent exploratory study found that while HPV vaccination did not significantly alter the vaginal microbiota, individuals with a *L. crispatus*-dominant microbiota exhibited lower local pro-inflammatory cytokine levels and a more balanced mucosal immune response—potentially facilitating better immunogenicity (Giraldo et al., 2021).

Furthermore, preclinical and early clinical studies suggest that the baseline composition of the vaginal microbiome may affect responsiveness to therapeutic vaccines targeting the E6/E7 oncoproteins of high-risk HPV types. For instance, responders to the PepCan therapeutic vaccine exhibited distinct microbiome signatures compared to non-responders, with enrichment of beneficial bacterial taxa and reduced presence of pro-inflammatory microbial metabolites (Ravilla et al., 2019). Innovative approaches involving genetically modified *Lactobacillus* strains as mucosal delivery vehicles for HPV antigens have shown promise in preclinical models. These lactic acid bacteria (LAB)-based vaccines leverage the gut-vagina axis to stimulate both systemic and mucosal immune responses. Their ability to induce localized CD8^+^ T cell responses in the genital tract makes them especially suitable for therapeutic or adjunctive vaccine strategies targeting persistent HPV infection and cervical intraepithelial lesions (Taghinezhad-S et al., 2021).

Despite the potential, disparities remain. Studies in women living with HIV have shown limited correlation between microbiota composition and vaccine-induced protection, highlighting the complexity introduced by immunosuppression and systemic factors (McClymont et al., 2022). Moreover, current vaccine platforms may not adequately account for interindividual variations in mucosal immunity shaped by microbiota, emphasizing the need for personalized or microbiota-informed vaccine strategies. Lastly, vaginal microbiome composition appears to modulate both innate and adaptive responses to HPV vaccination. Restoring microbial balance, through probiotics, prebiotics, or LAB-vectored antigens, could enhance vaccine efficacy, especially in populations at high risk of persistent HPV infection and CC (Huang et al., 2024; Ravilla et al., 2019; Taghinezhad-S et al., 2021).

## Microbiome-targeted therapeutic strategies in HPV-associated cervical cancer

Given the multifaceted role of the vaginal microbiota in modulating HPV persistence, local immunity, and epithelial integrity, strategies that target microbial dysbiosis are emerging as promising adjuncts or alternatives to conventional therapies in HPV-associated CC. These microbiome-modulating approaches include the use of probiotics, prebiotics, synbiotics, and genetically engineered bacterial vectors for vaccine delivery (Lin et al., 2010). Probiotics, particularly strains of *Lactobacillus crispatus*, *L. jensenii*, and *L. gasseri*, are being explored for their ability to restore vaginal homeostasis. These lactobacilli lower vaginal pH through lactic acid production, outcompete pathogenic bacteria, and exert anti-inflammatory effects. Clinical studies have shown that oral or vaginal administration of probiotic strains can enhance HPV clearance rates and improve cervical cytological outcomes (Huang et al., 2024; Li et al., 2020). Furthermore, *L. crispatus* has been shown to downregulate HPV oncogene expression and inhibit CC cell proliferation *in vitro* and *in vivo*, underscoring its potential therapeutic relevance (Huang et al., 2024). Prebiotics, such as inulin or glycogen-based formulations, support the selective growth of beneficial bacteria and may act synergistically with probiotics. Emerging formulations aim to enhance mucosal delivery and adherence, thereby maximizing colonization and immunomodulatory potential (Huang et al., 2024). In a more advanced direction, probiotic-vector HPV therapeutic vaccines have shown preclinical efficacy. These involve genetically modified *Lactobacillus* or *Lactococcus lactis* strains engineered to express HPV E6 and E7 oncoproteins, enabling mucosal delivery of antigens directly at the infection site. LAB-based vaccines stimulate both systemic and local cytotoxic T lymphocyte responses, offering a non-invasive, needle-free alternative to conventional vaccine delivery platforms (Huang et al., 2024; Lin et al., 2010). In parallel, attention has been drawn to the intratumoral microbiome, which may influence local immune responses and treatment resistance. Targeting the tumor-resident microbiota with engineered bacteria capable of delivering immunostimulatory molecules or checkpoint inhibitors is being evaluated as a novel form of bacterial immunotherapy (Mitra et al., 2015). Moreover, studies have proposed metabolic reprogramming as a therapeutic avenue. Microbial metabolites, such as D-lactic acid, have been shown to inhibit cervical stem cell self-renewal and slow tumor progression via pathways like PI3K-AKT and YAP1, suggesting that manipulation of microbial metabolic outputs could influence carcinogenic processes (Huang et al., 2024). Despite encouraging results, clinical implementation of microbiome-targeted therapies faces challenges, including interindividual variability in microbiota composition, optimal formulation and delivery methods, and regulatory oversight. Nevertheless, these strategies hold promise for complementing existing interventions and offer new hope in regions with limited access to traditional vaccines and screening programs (Hu and Ma, 2018; Li et al., 2020; Wu et al., 2024).

## Conclusions and future perspectives

Recent research has firmly established the vaginal microbiome as a critical player in the natural history of HPV infection and its progression toward cervical neoplasia. The predominance of *Lactobacillus* species, particularly *L. crispatus*, remains a hallmark of a healthy vaginal ecosystem, serving to suppress pathogenic colonization through acidification and metabolite secretion. Conversely, the shift toward diverse, non-*Lactobacillus*-dominant communities, such as those classified under Community State Type IV, has been consistently associated with increased HPV persistence, chronic genital inflammation, and elevated risk of cervical dysplasia and cancer (Norenhag et al., 2024; Ottinger et al., 2024; Łaniewski et al., 2020a).

Emerging metagenomic and multi-omics approaches have enabled a more granular understanding of these microbial ecosystems, revealing taxonomic, metabolic, and functional alterations that contribute to oncogenic microenvironments. For instance, an overrepresentation of *Gardnerella vaginalis*, *Fannyhessea vaginae*, and *Peptoniphilus lacrimalis* in dysplastic patients coincides with enriched genes in nucleotide and peptidoglycan biosynthesis, suggesting a metabolically reprogrammed microbiota that favors inflammation and epithelial transformation (Norenhag et al., 2024). Moreover, host-microbiome interactions extend beyond compositional changes, with increasing evidence that specific microbial patterns influence immune checkpoint expression, impacting the tumor-immune axis within the cervicovaginal milieu (Jung et al., 2025; Łaniewski et al., 2020a).

Despite these advances, several unresolved challenges persist. One critical limitation is the heterogeneity in study designs, sampling protocols, and sequencing technologies, which hampers cross-study comparisons and the development of clinically relevant biomarkers. Additionally, most current datasets are cross-sectional, thereby limiting the ability to infer causality between microbial states and cervical neoplastic progression (Huang et al., 2024; Kudela et al., 2021; Cheng et al., 2020). Longitudinal cohort studies with multi-timepoint sampling and integration of host, microbial, and immunological data are urgently needed to delineate temporal dynamics and causal relationships.

An important and underexplored dimension is the influence of sociodemographic and behavioral factors, such as race, contraceptive use, hygiene practices, and antibiotic exposure, on vaginal microbial composition. These variables may confound observed associations or represent modifiable targets for preventive interventions. For instance, the use of menstrual cups has been correlated with more stable *Lactobacillus*-dominant communities, suggesting the potential for non-pharmaceutical modulation of microbiota (Ottinger et al., 2024). Public health strategies that consider these determinants alongside vaccination and screening may offer synergistic benefits in reducing HPV-related disease burden.

From a therapeutic standpoint, efforts to restore or maintain *Lactobacillus* dominance through vaginal probiotics, live biotherapeutic products, or prebiotic interventions show promise but remain largely experimental. Early-phase trials suggest the feasibility of shifting CSTs via microbial interventions, yet their clinical utility in HPV clearance or cervical intraepithelial neoplasia (CIN) regression remains unproven (Mitra et al., 2020; Cheng et al., 2020; Ottinger et al., 2024). Furthermore, there is a pressing need for mechanistic validation in animal models or *ex vivo* systems that recapitulate the cervicovaginal microenvironment. Organoids and humanized vaginal microbiota murine models are emerging as powerful platforms to investigate how microbial metabolites and structural components modulate epithelial integrity, immune surveillance, and HPV oncogene expression (Li et al., 2020; Ottinger et al., 2024).

In addition, advances in immune-oncology highlight the microbiome’s capacity to modulate response to immunotherapies, particularly immune checkpoint inhibitors. While these associations are well-characterized in gastrointestinal and pulmonary cancers, recent findings suggest that vaginal microbiota composition influences the expression of PD-L1, TIM-3, and other checkpoint proteins in cervical neoplasia, potentially shaping the tumor microenvironment’s immunogenicity and responsiveness to therapy (Jung et al., 2025; Łaniewski et al., 2020a). As such, profiling the vaginal microbiota could eventually inform treatment stratification and predict response to emerging immunotherapies in cervical cancer.

Looking forward, several translational frontiers warrant prioritization. First, development of CST-based diagnostic and prognostic tools could revolutionize cervical cancer screening and triage, particularly in resource-limited settings. Vaginal microbiome profiles could augment current cytological and HPV-based algorithms, identifying women at highest risk of progression despite negative cytology or transient HPV infection. Second, integrating microbiome-targeted approaches with existing preventive strategies, such as HPV vaccination, could yield synergistic effects, particularly in populations with high prevalence of CST IV microbiota. Third, elucidating the bidirectional communication between the vaginal microbiome and host epigenetic, transcriptomic, and immunologic responses may uncover novel molecular targets for intervention.

In conclusion, the vaginal microbiome is no longer a passive bystander but an active determinant of HPV persistence and cervical oncogenesis. While recent discoveries have expanded our understanding of microbial composition and its functional roles, translating these insights into clinically actionable strategies remains a formidable yet promising challenge. Collaborative efforts across microbiology, gynecology, immunology, and systems biology will be essential to advance this frontier, ultimately contributing to personalized risk assessment, targeted prevention, and improved therapeutic outcomes in cervical cancer.
